# Is Endometrial Scratching Beneficial for Patients Undergoing a Donor-Egg Cycle with or without Previous Implantation Failures? Results of a Post-Hoc Analysis of an RCT

**DOI:** 10.3390/diagnostics11071167

**Published:** 2021-06-26

**Authors:** Alexandra Izquierdo, Laura de la Fuente, Katharina Spies, David Lora, Alberto Galindo

**Affiliations:** 1 Gynaecology Unit, Médipôle Hôpital Mutualiste Lyon-Villeurbanne, 69100 Villeurbanne, France; 2 Human Reproduction Unit, Department of Obstetrics and Gynaecology, University Hospital 12 de Octubre, Avda, Andalucia s/n, 28041 Madrid, Spain; lfuente@salud.madrid.org; 3 ProcreaTec–IVF Spain, Manuel de Falla 6, 28036 Madrid, Spain; katharina.spies.rojo@gmail.com; 4 Clinical Research Unit (imas12-CIBERESP), University Hospital 12 de Octubre, Avda, Andalucia s/n, 28041 Madrid, Spain; david@h12o.es; 5 Facultad de Estudios Estadísticos, Complutense University of Madrid, 28040 Madrid, Spain; 6 Fetal Medicine Unit—Maternal and Child Health and Development Network (Red SAMIDRD12/0026/0016), Department of Obstetrics and Gynaecology, 12 de Octubre Research Institute (imas12), University Hospital 12 de Octubre, Complutense University of Madrid, Avda, Andalucia s/n, 28041 Madrid, Spain; agalindo@salud.madrid.org

**Keywords:** endometrial scratching, in vitro fertilization, recurrent implantation failure, egg donor IVF cycle, endometrial receptivity

## Abstract

Endometrial scratching (ES) has been proposed as a useful technique to improve outcomes in in vitro fertilization (IVF) cycles, particularly in patients with previous implantation failures. Our objective was to determine if patients undergoing egg-donor IVF cycles had better live birth rates after ES, according to their previous implantation failures. Secondary outcomes were pregnancy rate, clinical pregnancy rate, ongoing pregnancy rate, miscarriage rate, and multiple pregnancy rate. We analysed the results of 352 patients included in the Endoscratch Trial (NCT03108157). A total of 209 were patients with one or no previous implantation failures (105 with an ES done in the previous cycle, group A1, and 104 without ES, group B1), and 143 were patients with at least two previous failed implantations (71 patients with ES, group A2, and 72 without ES, group B2). We found an improvement in pregnancy rates (62.9% in group A1 vs. 55.8% in group B1 vs. 70.4% in group A2 vs. 76.4% in group B2, *p* = 0.028) in patients with at least two previous implantation failures, but this difference was not statistically different when we compared clinical pregnancy rates (59.1% vs. 51.0% vs. 64.8% vs. 68.1% in groups A1, B1, A2 and B2, respectively, *p* = 0.104) and live birth rates (52.4% vs. 43.3% vs. 57.8% vs. 55.6% in groups A1, B1, A2 and B2, respectively, *p* = 0.218). According to these results, we conclude that there is no evidence to recommend ES in egg-donor IVF cycles, regardless of the number of previous failed cycles.

## 1. Introduction

In 2003, Barash et al. published for the first time their results on patients undergoing endometrial scratching (ES) before an in vitro fertilization (IVF) cycle. They aimed to enhance endometrial receptivity, as this had been seen in guinea pigs when an intentional injury was caused before embryo implantation [1]. They performed four endometrial injuries during the cycle prior to the embryo transfer and found an improvement in implantation rate (IR), pregnancy rate (PR), and live birth rate (LBR) in the study group [2]. Several studies were conducted after that and some authors reached the same conclusions [3,4], but others did not [5]. When only patients with recurrent implantation failure (RIF) were considered, ES seemed to have a better impact on the outcomes [6], as the review by Potdar et al. [7] and the Cochrane review by Nastri et al. showed in their results [8].

In 2019, Vitagliano et al. published a meta-analysis on the impact of ES in infertile women undergoing a first embryo transfer, and they could not find a difference between groups in terms of clinical pregnancy rates (CPR), miscarriage rate (MR), ongoing pregnancy rate (OPR), LBR, multiple pregnancy rate (MulPR), and ectopic pregnancy rate (EPR) in fresh or frozen embryo transfers [9]. They found a negative impact in one study that evaluated ES performed on the day of the oocyte retrieval [10]. The same authors published a meta-analysis of studies including patients with at least one previous failure, and found an increased LBR and CPR in patients who had two or more previous unsuccessful cycles [11]. In addition, a review conducted of studies that included patients who had at least one previous failed cycle found a significative increase in CPR and LBR after ES, but differences were not significative if studies with a high risk of bias were excluded [12]. Nevertheless, neither of these reviews included the last randomised controlled trial (RCT) published, the largest to date, that could not find, in their subgroup analysis of IVF patients, any difference in the LBR of patients undergoing a first cycle or after previous failed attempts [13]. 

The underlying mechanism that would lead to benefits of ES remains uncertain. Some authors have suggested that ES would lead to a better synchronization between embryo and endometrium through the endometrial maturation retardation [14], while others have advocated that ES would enhance decidualization through the induction of cytokine and growth factor secretion as well as immune cell invasion [15], or that ES would modify endometrial gene expression of specific factors involved in embryo implantation [16].

It is known that ovarian stimulation may induce an asynchrony between the embryo and the endometrium [17], due to the high oestrogen levels achieved during controlled ovarian stimulation, and this endometrial development is also different from one patient to another as it is determined by each woman’s ovarian reserve and stimulation protocols. In addition, embryo implantation potential may also be very different among patients undergoing IVF cycles as embryo quality and euploidy depend on maternal age [18]. With this background, we designed an RCT in egg donor recipients in order to assess the real effect of ES under similar endometrial conditions and with the highest embryo quality [19], including patients with and without previous implantation failures. Our results showed no differences in PR (65.9% vs. 64.2%, *p* = 0.823), CPR (61.4 vs. 58.0, *p* = 0.587) and LBR (54.5% vs. 48.3%, *p* = 0.286) between intervention and control groups in intention-to-treat (ITT) analysis.

The definition of RIF has been subject of debate during the last years as it is difficult to determine if a negative result is due to implantation deficient phenomena or to impaired embryo quality. This has led to many different definitions among authors, who may consider recurrent implantation failure from two to six previous failed embryo transfers, with fresh or frozen embryos [20]. In 2014, a thorough review of implantation failure definition and therapies defined RIF as the implantation failure of four or more good quality embryos, including fresh and frozen embryos, in at least three embryo transfers [21]. Nevertheless, recent meta-analyses and reviews consider RIF patients those who have undergone at least two previous embryo transfers, as they have found that, according to this definition, RIF patients had improved outcomes when an ES was performed [8,9,11]. The reduction in the total number of transferred embryos considered in the RIF definition is related to an increase in embryo selection due to laboratory culture conditions and pre-implantation genetic testing, reducing the impact of implantation failure associated with impaired embryo quality or embryo genetic conditions.

Thus, we performed a subgroup analysis of the outcomes of patients included in the Endoscratch Trial (NCT03108157), dividing our patients into RIF patients (with two or more previous failed implantations) and non-RIF patients (with a maximum of one previous failed embryo transfer) to determine the efficacy of ES in improving live birth rates in egg-donor IVF cycles according to previous implantation failures.

## 2. Materials and Methods

The overall number of patients included in the Endoscratch Trial (NCT03108157) was 352, of whom 176 were allocated to receive an ES in the luteal phase of the cycle preceding the embryo transfer cycle (group A), and another 176 to receive no intervention (group B). Sample size was calculated upon egg-donor IVF average pregnancy rates (60%) in order to detect a difference of 15% between the two groups.

We performed an analysis according to the number of previous failed embryo transfers, including own egg and donor egg, and fresh and frozen embryo transfers. This secondary analysis of our RCT was not included in the original design of the trial and thus sample size according to the number of implantation failure patients to be included in each arm was not calculated. Out of the 352 patients, 209 had a maximum of 1 previous failed embryo transfer (non-RIF patients), and 143 had at least two previous failed transfers (RIF patients). Among the 209 non-RIF patients, 105 belonged to group A (group A1) and received an ES, and 104 patients belonged to group B (group B1) and did not receive any intervention. Similarly, among the 143 RIF patients, 71 were included in group A (group A2) and 72 in group B (group B2).

ES was performed as described in the literature, with a biopsy catheter (Pipelle de Cornier, Laboratoire CCD, Paris, France) under abdominal ultrasound guidance [19]. No anaesthesia was required for the procedure and no major complications were reported. Those patients with a previous uterine intervention, such as ES or hysteroscopy, performed less than a month before the randomization were excluded from the RCT to avoid any possible effect on the subsequent embryo transfer (*n* = 27 patients excluded).

All egg donors underwent a conventional antagonist IVF protocol. Eggs were fertilized by ICSI (intracytoplasmic sperm injection) and embryos were kept in culture media until day 5 (blastocyst stage), when embryo transfer was performed. All recipients underwent endometrial preparation with oral (oestradiol valerate 6–10 mg) or transdermal oestrogens (oestradiol 100–200 mcg), and vaginal progesterone (400 mcg micronized progesterone every 12 h) was added 5 days before transfer. Treatment efficacy was determined by a transvaginal scan to check the endometrial pattern and thickness, and a blood test to determine oestradiol and progesterone levels on the day of the egg fertilization. Those patients with ovarian activity were prescribed an oral contraceptive pill to help synchronization with their donor, as well as GNRH analogues to induce ovarian quiescence.

A positive pregnancy test was considered if β-hCG levels were over 10 mUI/mL 12 days after embryo transfer. CPR was diagnosed if a vaginal ultrasound confirmed an intrauterine gestational sac 4 weeks after the embryo transfer (6th week of pregnancy). IR was calculated by the ratio between the number of gestational sacs observed and the number of replaced embryos. The birth of a living baby beyond the 24th week of pregnancy confirmed a live birth and all pregnancies lost before that moment were considered as miscarriages.

Statistical analysis was done using Stata 16 for Windows (StataCorp LP, College Station, TX, USA). We use mean and standard deviation to describe qualitative variables, frequency distribution to summarize qualitative variables and median and 25% and 75% centiles for non-normal variables. Outcomes were analysed with a chi-squared test or Fisher’s exact test.

The ES effect is described by absolute and relative frequencies, and the association strength is determined by raw risk ratio (RR) with 95% confidence intervals. A general linear model with a log link and binomial distribution was used to estimate the strength of association between outcomes, adjusted by independent variables. Results are presented as RR and 95% confidence intervals. Statistical significance was considered 0.05 (5% both sides α error) for all comparisons.

## 3. Results

All groups had similar baseline characteristics in terms of patient age, partner age, smoking habits, body mass index (BMI), race, and previous live births, as is shown in Table 1. However, patients with two or more previous implantation failures had a higher rate of previous miscarriages, including clinical and biochemical miscarriages.

Since all cycles considered were egg donor IVF cycles, similar endometrial and embryo quality parameters were expected. We found no differences in endometrial thickness (8.42 vs. 8.86 vs. 8.91 vs. 8.48 mm in groups A1, B1, A2 and B2 respectively, *p* = 0.164), nor in endometrial preparation duration (16.77 vs. 16.81 vs. 17.14 vs. 17.56 days in groups A1, B1, A2 and B2 respectively, *p* = 0.556). Hormonal parameters on the day of egg fertilization were also similar (Table 2).

Sperm parameters were also considered, as they may interfere in embryo quality, and total sperm count, total sperm A + B mobility, normal morphology, and total motile sperm count were comparable between the four groups as well (Table 3).

Regarding laboratory details, no differences were observed in donor age, the use of frozen or fresh eggs, the number of eggs obtained and fertilized, or the total number of embryos obtained. A mean of 1.25 and 1.26 embryos were replaced in groups A1 and B1, and 1.33 and 1.42 embryos in groups A2 and B2, respectively (*p* = 0.185), and a comparable number of extra embryos were frozen in each group after the embryo transfer (2.51, 2.93, 2.59, and 2.85 in groups A1, B1, A2 and B2, respectively, *p* = 0.399) (Table 4).

Embryo quality was also similar in the four groups, as is detailed in Table 5. 

We performed an ITT analysis and we found a lower PR in non-RIF patients (62.9% and 55.8% in non-RIF patients, groups A1 and B1, vs. 70.4% and 76.4% in RIF patients, groups A2 and B2, respectively, *p* = 0.028), but we could not find this difference when we performed a subgroup analysis of the results in the control and intervention groups in non-RIF patients (62.9% vs. 55.8% in groups A1 and B1, *p* = 0.262) and RIF patients (70.4% vs. 76.4% in groups A2 and B2, respectively, *p* = 0.419). In addition, we found no differences when we compared CPR among groups (59.1% vs. 51.0% vs. 64.8% vs. 68.1% in groups A1, B1, A2, and B2, respectively, *p* = 0.104), which confirms that the increase in PR was not clinically relevant. Finally, a total of 55/105 patients in group A and 45/104 patients in group B in non-RIF patients, and 41/71 patients in group A and 40/72 patients in group B in RIF patients had a live birth (52.4% vs. 43.3% vs. 57.8% vs. 55.6% in groups A1, B1, A2, and B2, respectively, *p* = 0.218), which meant no differences in terms of LBR among these populations either, as is detailed in Table 6.

Pregnancy complications and delivery details are summarized in Table 7 and showed no differences among groups.

## 4. Discussion

We designed the Endoscratch Trial to obtain information about ES effects in model conditions, as egg donor cycles offer results of homogeneous endometrial preparation, avoiding the bias of individual response to ovarian stimulation, and the best embryo quality, eliminating the impact of maternal age in the implantation potential.

Our post-hoc analysis found a higher PR in the group of RIF patients (subgroups A2 and B2) versus non-RIF patients (group A1 and B1), but this difference was not detected when we compared subgroups in the RIF-group (group A2 vs. group B2), which means that this increase is not associated with the ES. Accordingly, our study reveals that there is no difference in CPR and LBR when comparing patients according to whether they have had previous implantation failures or not, both in group A and B. These results are consistent with those of the largest study published in 2019 by Lensen et al. that included own-egg IVF cycles in RIF and non-RIF patients, and subgroup analysis did not find any differences between these groups regardless of whether ES was performed or not [13]. These results may raise doubt on the real ability of ES to increase the presence of cytokines required for implantation, and also to induce a long-lasting wound healing process that would go beyond the next menstruation and would persist until the following cycle where the embryo transfer takes place.

In addition, our results show that patients with RIF have a higher previous miscarriage rate than those who have not suffered previous implantation failures, which may mean that the former had an unknown underlying additional reason for these pregnancy failures.

Nevertheless, the possible benefits of ES remain a controversial issue as studies published to date on this subject are very heterogeneous, which entails a real problem when we try to estimate an overall effect. The first study by Barash et al. included RIF and non-RIF patients and found a two-fold increase in IR (27.7% vs. 14.2%, *p* = 0.00011), PR (66.7% vs. 30.3%, *p* = 0.00009), and LBR (48.0% vs. 23.6%, *p* = 0.016) in those patients who underwent four endometrial injuries during the cycle prior to the embryo transfer [2]. Similar results were obtained by other authors who performed several injuries to their patients [6], but also by authors who performed a single injury [3,4,22]. On the contrary, many other authors have not been able to find a difference either with one or more ES [5,23,24]. Regarding the moment of the cycle were the ES is performed, some studies show the results of ES performed in the follicular phase [25,26], and others in the luteal phase [3,4,24], or in both [2,5,13,23], obtaining different conclusions. The only study including the ES the day of the egg retrieval showed a decrease in IR, CPR and OPR [10]. Most studies have included a control group with no intervention [4,13], but some others have considered those undergoing a mock technique (such as a cervical pipelle inserted into the cervix), or a previous hysteroscopy [26] as control patients, which may have caused some kind of endometrial damage as well and could be considered a bias [23]. Regarding the target population of studies, some authors have included only first IVF cycles [4,27], some others only RIF patients [2,6,23,28], and others all patients without distinction [13,29], obtaining diverse results. Finally, the number of patients included in studies is very different as some studies had a low number of patients recruited [23], or were terminated before completion [29,30,31]. It is easy to understand that all these diverse criteria make it very difficult to analyse the real effect of ES. The last meta-analysis on seven studies with 1354 participants to assess the impact of ES in infertile women undergoing a first embryo transfer was published in 2019 by Vitagliano et al., and they could not find a difference between groups in terms of CPR, MR, OPR, LBR or MulPR. On the contrary, their meta-analysis of 10 studies including 1468 patients who had had at least one previous failure found an increased LBR (RR 1.38, 95% CI 1.05–1.80) and CPR (RR 1.34, 95% CI 1.07–1.67) in those patients with two or more previous failed embryo transfers [11]. Appendix A summarizes the main characteristics and outcomes of the studies mentioned. 

The main strength of our analysis is that it refers to an RCT including only egg recipients who had homogeneous endometrial preparations and who received good quality embryos. On the contrary, the main limitation is that this subgroup analysis was not considered at the initial study design and thus no randomized stratification was performed and no sample calculation for these subgroups was considered, which may lead to a limited power to establish robust evidence.

## 5. Conclusions

According to our results, based on the post-hoc analysis of an RCT, ES cannot be recommended as a strategy to improve clinical outcomes in patients undergoing egg donor IVF cycles, regardless of their previous failed treatments. Nevertheless, since our analysis concerns a low number of patients, these conclusions should be taken cautiously and larger studies in egg donor recipients targeting RIF patients should be performed.

## Figures and Tables

**Table 1:** Baseline characteristics of patients.

	Non-RIF Patients		RIF Patients		*p*
	Group A1	Group B1	Group A2	Group B2	
*n*	105	104	71	72	-
Age (years), mean (Sd)	41.59 (4.39)	41.73 (5.19)	40.85 (3.94)	41.17 (3.70)	0.667
Partner’s age (years), mean (Sd)	41.46 (6.66)	41.55 (7.64)	41.85 (5.97)	42.67 (6.95)	0.705
Body mass index (kg/m^2^), mean (Sd)	22.91 (3.30)	23.13 (3.48)	22.59 (3.53)	22.61 (3.06)	0.667
Smoking habit, *n* (%)	21 (20.00)	21 (20.19)	13 (18.31)	8 (11.11)	0.399
Race, *n* (%)					0.057
Arabian	10 (9.52)	5 (4.81)	3 (4.23)	2 (2.78)	-
Asian	1 (0.95)	1 (0.96)	0 (0.00)	3 (4.17)	-
Black	2 (1.90)	8 (7.69)	2 (2.82)	2 (2.78)	-
Caucasian	88 (83.81)	80 (76.92)	64 (90.14)	62 (86.11)	-
Other *	4 (3.81)	10 (9.62)	2 (2.82)	3 (4.17)	-
Previous live births, *n* (%)	14 (13.33)	19 (18.27)	14 (19.72)	17 (23.61)	0.362
Previous miscarriage **, *n* (%)	35 (33.33)	26 (25.00)	35 (49.30)	28 (38.89)	0.009

RIF: recurrent implantation failure. A1: intervention group of non-RIF patients, A2: intervention group of RIF patients, B1: control group of non-RIF patients, B2: control group of RIF patients. * Includes Hispanic and mixed races. ** Includes clinical and biochemical miscarriages.

**Table 2:** Endometrial preparation details.

	Non-RIF Patients		RIF Patients		*p*
	Group A1	Group B1	Group A2	Group B2	
*n*	105	104	71	72	0
Endometrial preparation duration (days), mean (Sd)	16.77 (3.58)	16.81 (3.71)	17.14 (3.89)	17.56 (4.58)	0.556
Endometrial thickness (mm), mean (Sd)	8.42 (1.65)	8.86 (1.89)	8.91 (1.99)	8.48 (1.77)	0.164
Blood oestrogen levels (ng/mL) *, mean (Sd)	866.89 (929.01)	860.15 (781.79)	877.58 (733.39)	911.00 (717.30)	0.980
Blood progesterone levels (ng/mL) *, mean (Sd)	0.56 (2.06)	0.33 (0.21)	0.34 (0.19)	0.39 (0.25)	0.464

RIF: recurrent implantation failure. A1: intervention group of non-RIF patients, A2: intervention group of RIF patients, B1: control group of non-RIF patients, B2: control group of RIF patients. * Before the start of progesterone supplementation.

**Table 3:** Sperm parameters.

	Non-RIF Patients		RIF Patients		*p*
	Group A1	Group B1	Group A2	Group B2	
*n*	105	104	71	72	-
Total sperm count (millions), mean (Sd)	126.02 (131.43)	120.65 (127.07)	132.30 (129.57)	120.14 (102.46)	0.923
Sperm motility A + B (%), mean (Sd)	44.80 (18.88)	45.49 (16.79)	40.68 (17.70)	44.94 (20.21)	0.346
Normal sperm morphology (%), mean (Sd)	4.17 (1.57)	4.33 (1.74)	4.01 (1.63)	4.38 (1.65)	0.514
Motile sperm count (millions), mean (Sd)	18.21 (21.59)	15.68 (17.82)	16.51 (25.98)	18.30 (25.13)	0.818

RIF: recurrent implantation failure. A1: intervention group of non-RIF patients, A2: intervention group of RIF patients, B1: control group of non-RIF patients, B2: control group of RIF patients.

**Table 4:** Laboratory details.

	Non-RIF Patients		RIF Patients		*p*
	Group A1	Group B1	Group A2	Group B2	
*n*	105	104	71	72	-
Egg donor’s age (years), mean (Sd)	24.11 (3.51)	24.96 (4.05)	25.64 (4.00)	24.89 (3.75)	0.072
Fresh eggs, *n* (%)	73 (69.52)	69 (66.35)	53 (74.65)	54 (75.00)	0.532
Number of mature eggs, mean (Sd)	8.67 (1.51)	8.98 (3.30)	8.81 (1.53)	8.63 (1.28)	0.690
Number of fertilized eggs, mean (Sd)	7.39 (1.78)	7.67 (3.25)	6.65 (2.39)	7.31 (1.84)	0.055
Number of embryos, mean (Sd)	4.93 (1.96)	5.19 (2.23)	5.04 (2.10)	5.36 (2.02)	0.569
Number embryos replaced, mean (Sd)	1.25 (0.50)	1.26 (0.51)	1.33 (0.56)	1.42 (0.52)	0.185
Patients without embryo transfer, *n* (%)	3.00 (2.86)	3.00 (2.88)	4.00 (5.63)	1.00 (1.39)	0.762
Number of frozen embryos, mean (Sd)	2.51 (1.72)	2.93 (2.20)	2.59 (1.96)	2.85 (2.00)	0.399

RIF: recurrent implantation failure. A1: intervention group of non-RIF patients, A2: intervention group of RIF patients, B1: control group of non-RIF patients, B2: control group of RIF patients.

**Table 5:** Embryo quality details.

	Non-RIF Patients				RIF Patients				*p*
	Group A1		Group B1		Group A2		Group B2		
*n*	105		104		71		72		
Embryo #1									0.762
Day 3 embryo									
Grade A, *n* (%)	4.00	3.81%	2.00	1.92%	1.00	1.41%	3.00	4.17%	
Grade B, *n* (%)	3.00	2.86%	1.00	0.96%	2.00	2.82%	0.00	0.00%	
Grade C, *n* (%)	1.00	0.95%	3.00	2.88%	0.00	0.00%	0.00	0.00%	
Day 5 embryo									
Morula, *n* (%)	0.00	0.00%	1.00	0.96%	1.00	1.41%	1.00	1.39%	
Early Blastocyst, *n* (%)	6.00	5.71%	5.00	4.81%	5.00	7.04%	3.00	4.17%	
Expanded Blastocyst, *n* (%)	16.00	15.24%	24.00	23.08%	16.00	22.54%	14.00	19.44%	
Hatching Blastocyst, *n* (%)	70.00	66.67%	65.00	62.50%	42.00	59.15%	49.00	68.06%	
Hatched Blastocyst, *n* (%)	2.00	1.90%	0.00	0.00%	0.00	0.00%	1.00	1.39%	
Embryo #2									0.694
Day 3 embryo	6.00	5.71%	3.00	2.88%	5.00	7.04%	3.00	4.17%	
Grade A, *n* (%)	3.00	2.86%	1.00	0.96%	1.00	1.41%	2.00	2.78%	
Grade B, *n* (%)	1.00	0.95%	1.00	0.96%	2.00	2.82%	1.00	1.39%	
Grade C, *n* (%)	2.00	1.90%	1.00	0.96%	2.00	2.82%	0.00	0.00%	
Day 5 embryo	24.00	22.86%	24.00	23.08%	23.00	32.39%	28.00	38.89%	
Morula, *n* (%)	2.00	1.90%	0.00	0.00%	0.00	0.00%	1.00	1.39%	
Early Blastocyst, *n* (%)	1.00	0.95%	5.00	4.81%	2.00	2.82%	3.00	4.17%	
Expanded Blastocyst, *n* (%)	8.00	7.62%	6.00	5.77%	10.00	14.08%	6.00	8.33%	
Hatching Blastocyst, *n* (%)	13.00	12.38%	12.00	11.54%	10.00	14.08%	18.00	25.00%	
Hatched Blastocyst, *n* (%)	0.00	0.00%	1.00	0.96%	1.00	1.41%	0.00	0.00%	

RIF: recurrent implantation failure. A1: intervention group of non-RIF patients, A2: intervention group of RIF patients, B1: control group of non-RIF patients, B2: control group of RIF patients.

**Table 6:** Cycle outcomes comparing RIF and non-RIF patients. Intention-to-treat analysis.

	Non-RIF Patients			RIF Patients			RIF vs. Non-RIF
	Group A1	Group B1	*p*	Group A2	Group B2	*p*	*p*
*n*	105	104	-	71	72	*-*	-
Positive pregnancy test, *n* (%)	66 (62.86)	58 (55.77)	0.262	50 (70.42)	55 (76.39)	0.419	0.028
Clinical pregnancy, *n* (%)	62 (59.05)	53 (50.96)	0.240	46 (64.79)	49 (68.06)	0.679	0.104
Miscarriage, *n* (%)	6 (5.71)	5 (4.81)	0.769	5 (7.04)	6 (8.33)	0.772	0.794
Ongoing pregnancy, *n* (%)	56 (53.33)	45 (43.27)	0.146	41 (57.75)	43 (59.72)	0.810	0.118
Live birth, *n* (%)	55 (52.38)	45 (43.27)	0.187	41 (57.75)	40 (55.56)	0.792	0.218
Multiple pregnancy, *n* (%)	5 (4.76)	8 (7.69)	0.381	8 (11.27)	8 (11.11)	0.976	0.338

RIF: recurrent implantation failure; A1: intervention group of non-RIF patients, A2: intervention group of RIF patients, B1: control group of non-RIF patients, B2: control group of RIF patients.

**Table 7:** Pregnancy complications and delivery details.

	Non-RIF Patients		RIF Patients		*p*
	Group A1	Group B1	Group A2	Group B2	
*n*	105	104	71	72	-
Biochemical pregnancy, *n* (%)	4 (3.81)	5 (4.81)	4 (5.63)	6 (8.33)	0.612
Early Miscarriage, *n* (%)	4 (3.81)	5 (4.81)	5 (7.04)	6 (8.33)	0.567
Late Miscarriage, *n* (%)	1 (0.95)	0 (0)	0 (0)	2 (2.78)	0.197
Abortion, *n* (%)	0 (0)	3 (2.88)	0 (0)	0 (0)	0.065
Ectopic pregnancy, *n* (%)	0 (0)	0 (0)	0 (0)	1 (1.39)	0.273
Preterm delivery *, *n* (%)	9 (8.57)	8 (7.69)	5 (7.04)	10 (13.89)	0.449
Caesarean section, *n* (%)	34 (32.38)	26 (25.00)	16 (22.54)	15 (20.83)	0.292
Instrumental delivery, *n* (%)	2 (1.90)	0 (0)	4 (5.63)	3 (4.17)	0.096
Gestational diabetes, *n* (%)	2 (1.90)	1 (0.96)	0 (0)	0 (0)	0.454
Pre-eclampsia, *n* (%)	4 (3.81)	2 (1.92)	0 (0)	4 (5.56)	0.197
Placentation anomalies, *n* (%)	1 (0.95)	0 (0)	0 (0)	0 (0)	0.501
Vanishing twin, *n* (%)	2 (1.90)	1 (0.96)	1 (1.41)	2 (2.78)	0.825
Cholestasis, *n* (%)	0 (0)	1 (0.96)	1 (1.41)	2 (2.78)	0.390
Retarded intrauterine growth, *n* (%)	0 (0)	1 (0.96)	0 (0)	0 (0)	0.495
2nd twin stillbirth, *n* (%)	0 (0)	1 (0.96)	0 (0)	1 (1.39)	0.542
Unknown, *n* (%)	1 (0.95)	1 (0.96)	1 (1.41)	2 (2.78)	0.737

RIF: recurrent implantation failure. A1: intervention group of non-RIF patients, A2: intervention group of RIF patients, B1: control group of non-RIF patients, B2: control group of RIF patients. * Preterm delivery was considered if birth happened before the 37th week of pregnancy.

## Data Availability

All data are available at ProcreaTec Fertility Clinic on demand. This trial was registered on 5 April 2017 as the ENDOSCRATCH Trial (NCT03108157) and protocol, statistical analysis plan, as well as main data and results are published.

## Appendix A

**Table A1:** Summary of main studies evaluating the effects of endometrial scratching in in vitro fertilization cycles.

Main Author	Year	*n*	Type of Trial	No. ES	Day of ES	Previous Failures	Control Group Intervention	Results
Barash et al. [2]	2003	135	Randomized prospective	4	Days 8 and 12 and 21 and 26	>4 cycles	NO	Significant increase in IR, CPR and LBR
Zhou et al. [3]	2008	121	Randomized prospective	1	Day 22	NO	NO	Significant increase in CPR. Non-significant increase LBR or IR
Karimzadeh et al. [22]	2009	115	Randomized prospective	1	Day 21 to 26	>2 cycles	NO	Significant increase in CPR
Karimzade et al. [10]	2010	156	Randomized prospective	1	OPU day	NO	NO	Significative reduction in IR, CPR and OPR
Baum et al. [23]	2012	36	Randomized prospective	2	Day 9 to 12 and day 21 to 24	>8 cycles	Cervical catheter	No significant differences in CPR or LBR
Shohayeb and El-Khayat [26]	2012	200	Randomized prospective	1	Day 4 to 7	>2 cycles	All patients underwent a previous hysteroscopy	Significant increase in IR, CPR and LBR
Nastri et al. [29]	2013	158	Randomized prospective	1	Day 14 to 21	0–4 cycles	NO	Significant increase in IR, CPR y LBR Finished with 158 patients due to positive results
Yeung et al. [5]	2014	300	Randomized prospective	1	Day 21	0–3 cycles	NO	No significant differences IR, CPR or LBR
Gibreel et al. [24]	2015	387	Randomized prospective	2	Days 21 and 24	0–4 cycles	NO	Significant increase in CPR and LBR if 2 or more previous failures
Seval et al. [25]	2016	345	Cohorts retrospective	1	Day 5 to 14	>2 cycles	NO	Significant increase in IR, CPR and OPR
Mahran et al. [4]	2016	418	Randomized prospective	1	Day 21 to 24	NO	NO	Significant increase in IR, CPR and LPR
Maged et al. [27]	2018	300	Randomized prospective	1	Day 21	NO	NO	Significant increase in IR and CPR
Frantz et al. [30]	2019	358	Randomized prospective	1	Day 21 to 24	0–1 cycles	NO	Significative reduction in IR, CPR and OPR. Terminated before completion due to bad results in ES group
Hilton et al. [31]	2019	332	Randomized prospective	1	Day 20 to 25	NR	NO	No differences in IR, CPR or LBR. Terminated before completion due to slow recruitment
Lensen et al. [13]	2019	1364	Randomized prospective	1	Day 3 to day 3 of transfer cycle	0–4 cycles	NO	No differences in PR, CPR, OPR, LBR, MR or MulPR
Bar et al. [6]	2020	300	Cohorts retrospective	2	Day 8 to 12 and day 19 to 23	>2 cycles	NO	Significant increase in PR, IR, CPR and LBR. Significative reduction in MR

ES: endometrial scratching. No. ES: number of injuries performed. Day of ES: days of the previous cycle when ES was performed. PR: pregnancy rate, IR: implantation rate, CPR: clinical pregnancy rate, OPR: ongoing pregnancy rate, MR: miscarriage rate, LBR: live birth rate, MulPR: multiple pregnancy rate. OPU day: ovum pick-up day.
